# Risk factors and influence of carbapenem exposure on the development of carbapenem resistant *Pseudomonas aeruginosa* bloodstream infections and infections at sterile sites

**DOI:** 10.1186/s40064-016-2438-4

**Published:** 2016-06-17

**Authors:** Michelle A. Barron, Kris Richardson, Meghan Jeffres, Bruce McCollister

**Affiliations:** Division of Infectious Diseases, University of Colorado Denver, 12700 E. 19th Ave, B168, Aurora, CO 80045 USA; Department of Clinical Pharmacy, Skaggs School of Pharmacy and Pharmaceutical Science, University of Colorado Denver, Mail Stop C 238, 12850 E. Montview Blvd, V20-1212, Aurora, CO 80045 USA

**Keywords:** Antibiotic resistance, *Pseudomonas aeruginosa*, Carbapenem resistance

## Abstract

**Background:**

Patients with *Pseudomonas aeruginosa* infections from blood or sterile sites were evaluated to determine risk factors associated with carbapenem resistance (CRPA) compared to carbapenem sensitivity (CSPA) as well as prior carbapenem use and the development of resistance.

**Findings:**

Retrospective chart review of 80 patients hospitalized with a documented *P. aeruginosa* infection during 2010–2011. Stored isolates were retested with both Kirby–Bauer disk diffusion and E-tests. Clinical characteristic of patients in the CRPA (N = 21) and the CSPA (N = 59) groups were similar. Hospital acquired (HA) infections were more common in the CRPA group compared to the CSPA group (71 vs 44 %, p = 0.04) and CRPA patients were more likely to have a Foley catheter at the time of infection (71 vs 37 %, p = 0.01). There was more carbapenem use in the CRPA group prior to onset of infection (59 vs 22 %, OR 5.1, 95 % CI 1.3–20.8, p = 0.01). Length of stay was significantly longer in the CRPA group (mean 44 days) compared to the CSPA group (mean 23 days), p = 0.02. Mortality between the two groups was similar and there were no differences between groups for death attributable to *Pseudomonas*.

**Conclusions:**

Patients with CRPA were more likely to have HA infections and to have a multidrug resistant profile. Other identifiable risks included a Foley catheter in place at the time of infection and exposure to a carbapenem prior to infection. Prompt removal of devices and judicious use of antibiotics may be interventions that can impact the development of this kind of infections.

## Background

Risk factors for developing a *Pseudomonas aeruginosa* blood stream infection include the presence of a urinary device, antimicrobial use within 30 days and the presence of a central venous catheter (CVC) (Schechner et al. 2009). Identifying risk factors for multi-drug resistant *P. aeruginosa* is especially of interest due to the limited number of agents that have good activity against this organism.

The University of Colorado Hospital (UCH) is a tertiary care academic medical center. Antibiogram data has been used to make adjustments to the hospital’s formulary. Fluoroquinolones became restricted house-wide in 2004 due to increased resistance. Ertapenem was added to the formulary in November 2004. Imipenem was removed from the formulary in 2007 and meropenem in 2010. Doripenem was introduced to the UCH formulary in November 2009.

*In vitro*, incubation of *P. aeruginosa* in media containing ertapenem can select for resistance; however, this occurred at concentrations higher than what is typically seen free in the serum of patients (Livermore et al. 2005). Several studies evaluating if previous exposure to ertapenem leads to the development of resistance to group 2 carbapenems in *P. aeruginosa* did not find an association (Lima et al. 2009; Carmeli et al. 2011; Eagye and Nicolau 2011); however, these studies did show an increased incidence of group 2 carbapenem resistance with previous exposure to group 2 carbapenems, aminoglycosides and penicillins. Most of these studies evaluate antibiotic usage (DOT and DDD) as the correlate to patient exposure to these antibiotics rather than at the individual level. Via a retrospective chart review, we sought to evaluate the contribution, if any, of prior carbapenem use in individual patients that developed *P. aeruginosa* infections in blood or sterile sites.

## Methods

### Chart review

This study was approved by our local institutional review board. A retrospective chart review of patients hospitalized at UCH with a documented *P. aeruginosa* blood stream infection or *P. aeruginosa* from another sterile site (cerebral spinal fluid, pleural fluid, peritoneal fluid, bone, or surgically obtained tissue) identified between 2010 and 2011 was undertaken. The point of reference for the data abstraction is the identification of the hospital stay associated with the *P. aeruginosa* infection from January 1, 2010 through December 31, 2011. *P. aeruginosa* susceptibility versus resistance was based on the on the susceptiblity reported at the time the clinical isolate was obtained. If unavailable at initial review, susceptibility or resistance was then classified based on disk diffusion testing. Antibiotic utilization data were obtained from the UCH pharmacy records. Classification of infections were defined as community-acquired, healthcare associated, or hospital acquired based on standard infection control surveillance definitions.

### Bacterial isolates

A total of forty-four clinical isolates of *P. aeruginosa* recovered from bloodstream infections in 2010–2011 were available for microbiological analysis. Only non-duplicate isolates per patient were included. *P. aeruginosa* ATCC strain 27,853 was used for quality control. All clinical isolates had been stored at −80 °C prior to use.

### Antimicrobial susceptibility tests

Frozen *P. aeruginosa* isolates were serially passaged two times on blood agar prior to performing susceptibility studies. Disk diffusion (DD) testing was performed as recommended by the Clinical and Laboratory Standards Institute (CLSI) (2009). The results were interpreted according to CLSI parameters (CLSI 2013).

Minimal inhibitory concentrations to imipenem, meropenem and doripenem were additionally determined for each of the *P. aeruginosa* isolates by the E-test method according to the manufacturer’s instructions (AB bioMérieux, Marcy l’Étoile, France). MIC values were determined manually and were interpreted according to 2013 CLSI parameters.

### Data management and statistical analysis

Study data was collected and managed using REDCap™. Risks for sensitive versus carbapenem resistance phenotypes were examined using descriptive statistics. The Mann–Whitney U test was used to compare risk factors amongst the two groups. The Fisher Exact test was used to test for association the disease-exposure association for each factor. The odds ratio and 95 % confidence interval were calculated.

Variables that were statistically different between carbapenem resistant and carbapenem sensitive groups were manually entered into a logistic regression model. Variables with a p value <0.05 were retained in the final multivariable model. SPSS version 22.0 (IBM Corp. Armonk, NY) was used for logistical regression analysis.

## Findings

Eighty patient charts were reviewed with 59 patients with carbapenem sensitive *P. aeruginosa* (CSPA)—32 blood isolates and 27 from sterile sites, and 21 with carbapenem resistant *P. aeruginosa* (CRPA)—15 blood isolates and 6 from sterile sites. Seventy six percent of the CRPA isolates were multi-drug resistant (≥3 classes of antibiotic resistance). Demographics and clinical characteristics of the patients were similar except for more solid organ transplant recipients in the CRPA group (21 vs 7 %, p = 0.04) than in the CSPA group (Table 1). The majority of the infections were either hospital acquired or healthcare associated with more hospital acquired infections in the CRPA group (71 vs 44 %, p = 0.04) (Table 1). However, the CSPA group were more likely to have infection healthcare associated infection with hospitalization of at least 30 days prior to current hospital admission (32 vs 10 %, p = 0.045). Patients with CRPA were more likely to have a Foley catheter in place at the time of infection (71 vs 37 %, p = 0.01) but there was no difference for other invasive devices including central venous catheter, urinary conduit, nephrostomy tubes, or nasogastric tubes. Fifty-eight (73 %) of the patients received at least one dose of antibiotics (19 received a carbapenem) prior to developing their *Pseudomonas* infection. There was more carbapenem use in the CRPA group prior to the onset of infection (59 vs 22 %, OR 5.1, 95 % CI 1.3–20.8, p = 0.01) (Table 1).

**Table 1:** Epidemiologic and clinical characteristics of 80 patients with *P. aeruginosa* infection from blood or sterile isolates

Variable	Carbapenem sensitive isolates (N = 59)	Carbapenem resistant isolates (N = 21)	p value
Age (years)			
Median (range)	55 (22–88)	57 (26–77)	0.86
Gender			
Male (%)	66	52	0.30
Race			
Caucasian (%)	81	86	0.75
African American (%)	12	14	0.69
Ethnicity			
Hispanic (%)	12	10	1.00
Type of acquisition			
Hospital acquired (%)	44	71	0.04
Health care associated (%)	42	24	0.19
Community acquired (%)	14	5	0.43
Underlying co-morbidities			
Chronic renal insufficiency (%)	19	24	0.75
Coronary artery disease (%)	15	24	0.52
Diabetes mellitus (%)	27	38	0.41
Hematologic malignancy (%)	14	14	1.00
Neutropenia (%)	14	14	1.00
Solid tumor (%)	20	19	1.00
Solid organ transplant (%)	7	21	0.04
Device use			
Central venous catheter (%)	54	76	0.12
Foley catheter (%)	37	71	0.01
Antibiotic use			
Prior antibiotics (%)	69	80	0.80
Prior carbapenem use (%)	22	59	0.01
Length of stay			
Mean days (range)	23 (1–127)	44 (4–223)	0.02

**Fig. 1:**
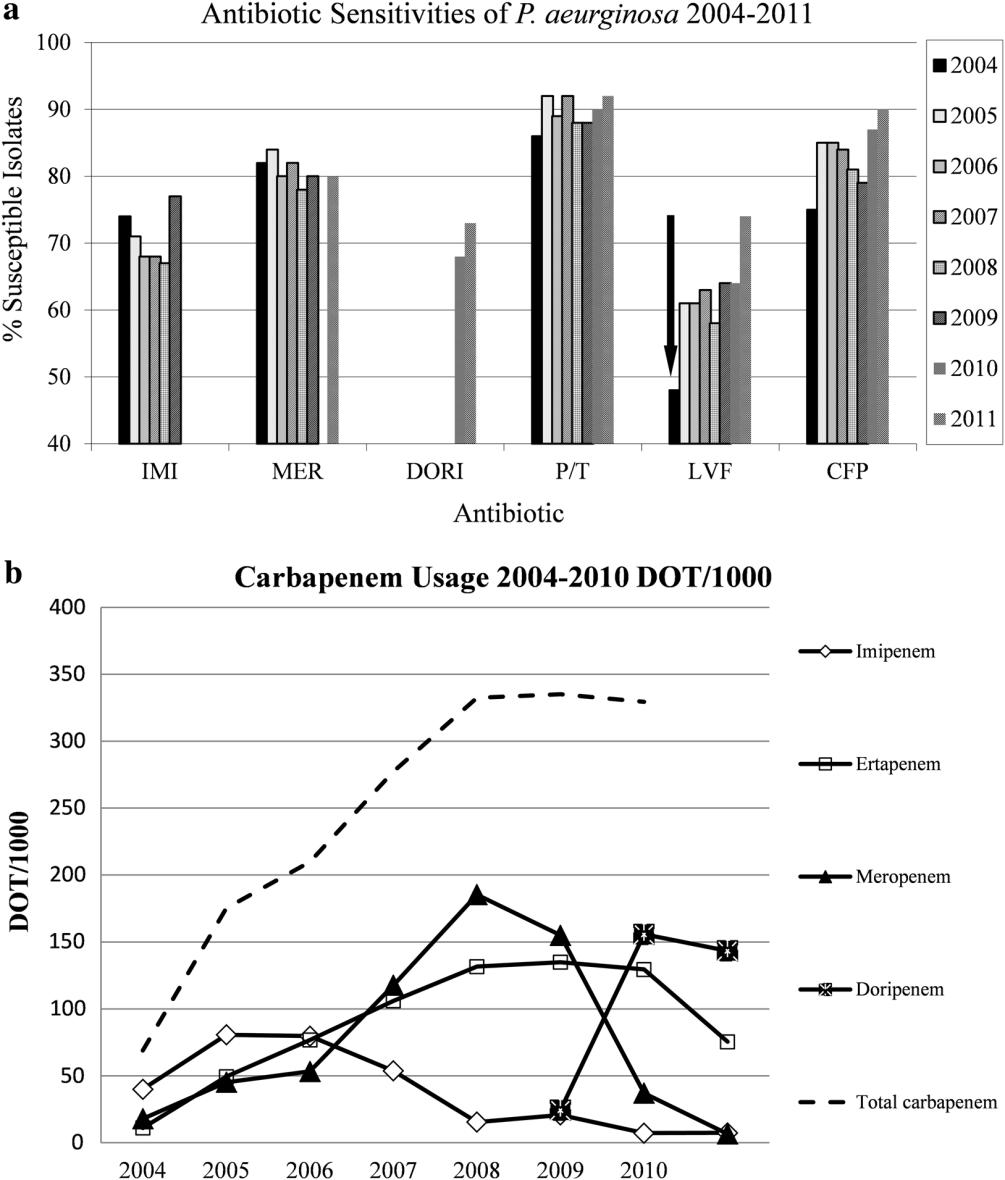
**a** Antibiogram for *P. aeruginosa* isolates at UCH from 2002 to 2011. Arrow denotes implementation of house-wide restriction of fluoroquinolones. *P*/*T* piperacillin/tazobactam, *GNT* gentamicin, *IMI* imipenem, *MER* meropenem, *DORI* doripenem, *CTX* ceftazidime, *CIP* ciprofloxacin. **b** Antibiotic usage at UCH from 2004 to 2011. There was a significant decrease of fluoroquinolones after restriction in 2004

Despite overall improvement in our antibiotic sensitivities to *P. aeruginosa* isolates, the sensitivities for carbapenems has not improved proportionately compared to the other agents (Fig. 1a). During this time, overall carbapenem use was increased (Fig. 1b). However, there was no association between CRPA and individual exposure to ertapenem, meropenem, imipenem, or doripenem or between group 1 and 2 carbapenems up to 30 days prior to the development of the infection. In addition, there was no statistical association with prior use of other antibiotics except for a trend towards more fluoroquinolone exposure in the CRPA group (47 vs 20 %, OR 3.7, 95 % CI 0.92–15.1, p = 0.05). Length of stay was significantly longer in the CRPA group (mean 44 days) compared to the CSPA group (mean 23 days), p = 0.02 (Table 1). Mortality between the two groups was similar and there were no differences between the groups for death attributable to *Pseudomonas*.

Logistic regression was done for all statistically significant variables between groups. Variables retaining statistical significance for carbapenem resistant infections were presence of a Foley catheter, transfer from another unit, prior carbapenem use, prior ertapenem use, prior fluoroquinolone use, and prior ciprofloxacin use (Table 2). To avoid redundancy between prior carbapenem and prior ertapenem use, only one variable was entered into the multivariate analysis at a time. The same technique was used for prior fluoroquinolone and prior ciprofloxacin use. Due to multiple iterations of the multivariate analysis, the odds ratios, 95 % confidence intervals, and p values closest to significance are reported in Table 2 as Adjusted. None of the variables maintained significance in the multivariate analysis.

**Table 2:** Logistic regression of factors associated with carbapenem-resistant *P. aeruginosa* infections

Factor	Unadjusted		Adjusted	
	Odds ratio (95 % CI)	p value	Odds ratio (95 % CI)	p value
Foley catheter	4.2 (1.4–12.4)	0.009	2.8 (0.8–9.5)	0.091
Transfer from another unit	5.4 (1.5–19.6)	0.010	4.1 (0.9–17.4)	0.056
Prior carbapenem use	5.9 (1.7–20.8)	0.005	2.8 (0.7–12.7)	0.168
Prior ertapenem use	11.5 (2.7–49.3)	0.001	4.8 (0.9–25.1)	0.059
Prior fluoroquinolone use	3.9 (1.2–12.4)	0.020	3.4 (0.8–14.6)	0.103
Prior ciprofloxacin use	6.7 (1.1–39.8)	0.036	3.3 (0.4–29.7)	0.280

Stored isolates were available for 44 patients with blood stream infections. Based on documentation in the clinical chart, 28 isolates were reported as sensitive to doripenem, 5 were intermediate, and 6 isolates were reported as resistant. Five isolates did not have sensitivities reported in the clinical chart at the time they were originally obtained. Subsequent testing via E-test revealed a good correlation with the initial DD results; however, 7 individual isolates (23 %) were reported as susceptible to doripenem (2), meropenem (1) or imipenem (4) based on DD but were considered intermediate susceptible based on their respective MICs. Susceptibility to one carbapenem did not always predict susceptibility to one of the other carbapenems. Based on MICs, 11 (37 %) doripenem susceptible isolates were intermediate or resistant to meropenem and imipenem and conversely, 6 (20 %) imipenem intermediate or resistant isolates were found to be susceptible to meropenem and doripenem.

## Discussion

Studies evaluating the influence of group 1 versus group 2 carbapenems on *P. aeruginosa* susceptibilities (Lima et al. 2009; Carmeli et al. 2011; Eagye and Nicolau 2011) primarily utilized antibiotic prescription data to determine associations between usage and subsequent resistance. In this study, while antibiotic usage was evaluated, patient level risk factors were assessed to determine any associations with the susceptibility patterns of the *P. aeruginosa.* In our study, patients with CRPA were more likely to have a hospital acquired infection and to have a multidrug resistant profile. Other identifiable risks included a Foley catheter in place at the time of infection, transfer from another unit, exposure to a carbapenem prior to infection including a trend towards an association with ertapenem usage, and prior fluorquinolone use (in the unadjusted model). This is noteworthy in that prior exposure to ertapenem has not been associated with subsequent carbapenem resistance; however, in the adjusted linear regression model, these risk factors were not statistically significant.

In a case control study by Lautenbach et al. (2006), patients with imipenem resistant *P. aeruginosa* were compared to those with imipenem sensitive isolates from all clinical sites including respiratory tract, urinary tract, skin or soft tissue, blood, abdomen, catheter, and other sites. The authors found that the only independent risk factor for imipenem resistance was prior FQ use. In addition, imipenem resistance was associate with longer length of hospital stay and increased mortality. We found a similar association with FQ use, but did not see the association with increased mortality; however, this may be a factor of a much smaller sample size in our study compared to the aforementioned study.

Fortaleza et al. (2006) evaluated risk factors associated with imipenem resistant *P. aeruginosa* in patients hospitalized in Brazil. One hundred and eight patients were evaluated and statistically significant risk factors for acquisition of imipenem resistant *P. aeruginosa* were previous admission to another hospital, hemodialysis, and therapy with imipenem, amikacin, and/or vancomycin. Our study did not find similar associations, though again, our sample size was likely underpowered to detect these differences.

In evaluation of outcomes, length of stay was longer in the CRPA group. There were 5 deaths in the CRPA group with 3 deaths (60 %) attributed to the *Pseudomonas* infection and 16 total deaths in the CSPA with 9 of the deaths (56 %) attributed to *Pseudomonas* infection. Four patients in the CRPA group received empiric therapy with a carbapenem and 2 of those patients died due to on-going sepsis; however, there was no difference in 30-day mortality between the two groups.

MIC testing of isolates revealed discordant results when compared to DD testing in a small percentage of cases and susceptibility to one carbapenem did not necessarily correlate with susceptibility or resistance to the other carbapenems tested. Limits of the study include retrospective nature and small sample size.

In conclusion, patients should be evaluated for risk factors for the development of resistant Pseudomonas including use of devices, recent hospitalization, transfer between hospital units and prior antibiotic use including the use of FQs and carbapenems. Prompt removal of devices and an aggressive antibiotic stewardship programs may help prevent the development and complications associated with CRPA.
